# Use of Verbal Autopsy to Determine Underlying Cause of Death during Treatment of Multidrug-Resistant Tuberculosis, India

**DOI:** 10.3201/eid2403.171718

**Published:** 2018-03

**Authors:** Poonam Ramesh Naik, Patrick K. Moonan, Abhay Subhashrao Nirgude, Hemant Deepak Shewade, Srinath Satyanarayana, Pracheth Raghuveer, Malik Parmar, Chinnappareddy Ravichandra, Anil Singarajipura

**Affiliations:** Yenepoya Medical College, Yenepoya University, Mangalore, India (P.R. Naik, A.S. Nirgude, P. Raghuveer);; Centers for Disease Control and Prevention, Atlanta, Georgia, USA (P.K. Moonan);; International Union Against Tuberculosis and Lung Disease, New Delhi, India (H.D. Shewade, S. Satyanarayana);; World Health Organization Country Office for India, New Delhi (M. Parmar);; National Tuberculosis Institute, Bangalore, India (C. Ravichandra);; Department of Health and Family Welfare, Government of Karnataka, Bangalore (A. Singarajipura)

**Keywords:** Multidrug-resistant tuberculosis, MDR TB, deaths, verbal autopsy, underlying cause of death, tuberculosis and other mycobacteria, India, bacteria

## Abstract

Of patients with multidrug-resistant tuberculosis (MDR TB), <50% complete treatment. Most treatment failures for patients with MDR TB are due to death during TB treatment. We sought to determine the proportion of deaths during MDR TB treatment attributable to TB itself. We used a structured verbal autopsy tool to interview family members of patients who died during MDR TB treatment in India during January–December 2016. A committee triangulated information from verbal autopsy, death certificate, or other medical records available with the family members to ascertain the underlying cause of death. For 66% of patient deaths (47/71), TB was the underlying cause of death. We assigned TB as the underlying cause of death for an additional 6 patients who died of suicide and 2 of pulmonary embolism. Deaths during TB treatment signify program failure; accurately determining the cause of death is the first step to designing appropriate, timely interventions to prevent premature deaths.

*Mycobacterium tuberculosis* resistant to >2 of the most potent TB drugs, isoniazid and rifampin, is classified as multidrug-resistant tuberculosis (MDR TB). Worldwide, an estimated 580,000 MDR TB cases emerge annually (*1*). Unfortunately, there are substantial gaps in MDR TB detection and treatment. Approximately 1 of 5 persons needing MDR TB treatment actually receive it, and among those who do receive treatment, less than half (48%) who start treatment finish successfully (*1**,**2*). These rates are driven by treatment failure, loss to follow-up, and premature death. In 2016, the proportion of deaths during MDR TB treatment in India was higher than the global average (20% vs. 14%) (*3*).

India follows the routine surveillance and reporting guidelines recommended by the World Health Organization (WHO) and considers any death that occurs during TB treatment as a TB-related death. Several studies have used all-cause mortality as a surrogate marker of mortality attributable to TB (*4**–**6*). This method of attributing all-cause mortality can overestimate TB case-fatality rates. Accurately determining the cause of death is the first step to designing appropriate and timely interventions to prevent premature deaths.

In settings with no or poorly documented vital registration and medical certification of the cause of death, verbal autopsy can be an essential public health tool for obtaining a reasonable estimation of the cause structure of mortality (*7*). Verbal autopsy uses systematic retrospective inquiry of family members about the symptoms and signs of illness before death to help determine the putative medical cause of death (*8*). The demand for and use of verbal autopsy data has rapidly gained importance and has been used to set global health priorities (*9**,**10*). Verbal autopsy data may improve surveillance and program monitoring and evaluation and could stimulate change in public health policy (*11**–**13*). In our study, we used the verbal autopsy method to determine the underlying causes of death for persons who died during MDR TB treatment (Technical Appendix).

## Methods

### Study Design and Population

We conducted a cross-sectional study of patients who died during MDR TB treatment during January–December 2016 at 3 drug-resistant TB (DR-TB) treatment centers in southwestern Karnataka state in India (Figure). The DR-TB centers serve 3 major populations of Karnataka (Bangalore, 17.9 million persons from 7 districts; Mangalore, 4.6 million persons from 3 districts; and Mysore, 10.1 million persons from 6 districts) (*3*). DR-TB centers, established with the support of Revised National Tuberculosis Control Program of India (RNTCP), are located within tertiary care centers, public hospitals, and teaching hospitals and follow national guidelines for the treatment of drug-resistant TB (*2*). The study participants included family proxies of deceased patients. For the purpose of data collection, family proxies included spouses, parents, adult children, siblings, or relatives involved in providing care to the deceased person.

**Figure:**
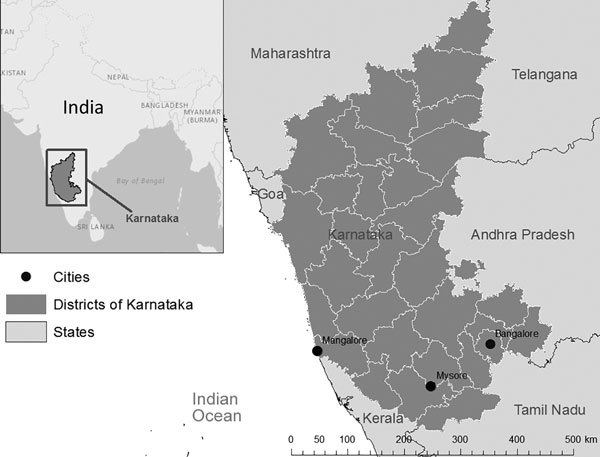
Locations of the 3 drug-resistant TB treatment centers in the state of Karnataka, India. Inset shows location of Karnataka in India.

MDR TB patients were started on a standardized second-line treatment regimen at DR-TB centers followed by ambulatory care in the community. Pretreatment clinical evaluations were required; these included chest radiograph; complete hemogram; liver, renal and thyroid function tests; HIV serology; screening for diabetes mellitus; calculation of body mass index; and for women, pregnancy tests. All patients were required to complete >7 days of inpatient treatment at DR-TB centers, where they began treatment for MDR TB in accordance with national guidelines (i.e., 6 months of kanamycin, levofloxacin, ethionamide, pyrazinamide, ethambutol, and cycloserine followed by 18 months of levofloxacin, ethionamide, ethambutol, and cycloserine). Patients who did not achieve culture conversion at 6 months, or who experienced culture reversion at any time during treatment, were considered to have poor treatment response and required reevaluation and second-line drug susceptibility testing. Throughout treatment, community-based directly observed treatment (DOT) providers monitored patients for adverse drug effects. DOT providers referred patients experiencing any major adverse drug reactions to appropriate health facilities. The management of existing comorbidities such as diabetes, liver or renal disease, and neurological or psychiatric disorders is also recommended by the RNTCP (*2*).

In accordance with national policy, district TB officers should conduct an audit for all deaths occurring among MDR TB patients to better understand potential programmatic gaps in TB diagnosis and treatment (*2*). However, TB death audits do not look into the underlying cause of death. 

We developed a semistructured questionnaire as a verbal autopsy tool to elicit information about behavioral, demographic, socioeconomic, and comorbid conditions; detailed symptoms; and chronology of events preceding death, with the most recent condition first and the earliest (e.g., the condition that started the sequence of events between normal health and death) last. We also abstracted relevant clinical information from medical records and, when available from the family, the cause of death as documented on a death certificate. We abstracted the information on date of death and the date of initiation of treatment from programmatic management of drug-resistant TB treatment cards to calculate the median duration of MDR TB treatment. A committee of 3 clinicians independently reviewed all available evidence to ascertain the underlying cause of death following national guidelines (*14*). A final assignment of the cause of death required the concurrence of >2 committee members. If 2 reviewers did not concur on cause of death, then they would meet in person to discuss their disagreement. The committee assigned the underlying cause of death as TB if it had initiated the sequence of illness events leading directly to death (*14*). If the underlying cause of death was not TB, then the committee also recorded what was believed to be the specific cause of death using codes from the International Classifications of Diseases, 10th Revision, as recommended by WHO (*15*). For patients with a cause of death not directly attributed to TB but that may have resulted from TB or TB treatment (e.g., drug-induced psychosis leading to suicide), we listed TB as an underlying cause of death.

### Data Collection

We conducted proxy interviews during December 2016–March 2017. The principal investigator, who had prior experience in conducting verbal autopsy, trained a team of investigators to obtain clinical histories and document the sequence of events preceding death using the verbal autopsy tool. The field investigators prepared participants psychologically for the verbal autopsy process and provided appropriate grief counseling when needed. The principal investigator supervised the field investigators to ensure correct protocol implementation, made home visits to identify potential challenges, provided feedback, and recommended corrective action to ensure quality data collection.

Families with an annual income <10,000 Indian rupees are eligible for government supplemental support with Below Poverty Line (BPL) ration cards (*16*). We used BPL cards as a proxy for socioeconomic status of the families.

We included history of smoking and alcohol consumption of the deceased person in the proxy questionnaire. We did not quantify the number of cigarettes smoked, nor the amount of alcohol consumed, because these details were deemed not accurate through proxy.

### Data Analysis

We used EpiData (EpiData Association, Odense, Denmark) to double enter, validate, and analyze quantitative data. We calculated simple proportions of selected demographic, behavioral, and clinical characteristics among cases with and without TB as the underlying cause. We used the χ^2^ test to detect differences in proportions among cases with and without TB as the underlying cause of death and the Fisher exact test when individual cell counts were <5 (α<0.05). We calculated the duration of MDR TB treatment as the number of days from date of initiation of treatment to date of death. We abstracted missed doses of TB drugs from treatment cards and used medians and interquartile ranges (IQRs) to describe the number of doses missed and duration of MDR TB treatment. We compared medians between cases with and without TB as the underlying cause of death using a Mann-Whitney test.

### Ethics Considerations

We obtained approval for the study protocol from the Yenepoya University Ethics Committee (Mangalore, India); Ethics Advisory Group of International Union Against Tuberculosis and Lung Disease (Paris, France); National Tuberculosis Institute (Bangalore, India); and US Centers for Disease Control and Prevention (Atlanta, GA, USA). We conducted proxy consent and verbal autopsies in the local language, Kannada. We ensured strict privacy and confidentiality during all participant encounters. All interviews occurred at participants’ homes at a time convenient to them. Institutional-, district-, and state-level authorities granted appropriate permission before initiating the study.

## Results

A total of 75 TB-related deaths occurred during the study period. For these, 72 family proxies (96%) consented to verbal autopsy. Most of the proxies were the spouse of the deceased (37%), followed by parents (16%), adult children (16%), siblings (16%), in-laws (10%), and other relatives (5%). In addition to verbal autopsy data available for 72 of the deceased patients, medical records for 8 patients were available from the family, and 4 patients had death certificates that their proxies provided during interviews. The death audit report was available for 2 patients.

The committee reviewed all available data and independently and unanimously assigned TB as the underlying cause of death for 49 (68%) patients. For the remaining 23 patients, the committee reached consensus after face-to-face deliberation (Table 1). The committee members assigned the underlying cause of death for 71 out of 72 patients. For 1 patient, there was insufficient evidence to assign a specific cause of death; we excluded this patient from further analysis. TB was the underlying cause for 77% (55/71) of the deaths (Table 2) (*14*). After extensive deliberations and discussions, the committee deemed TB or TB treatment the underlying cause of death for 6 patients who died of suicide. Suicidal ideation may be associated with TB because of psychological stress, social stigma, hopelessness, and depression caused by MDR TB diagnosis or treatment; in addition, cycloserine used to treat MDR TB may have caused psychosis or depression resulting in suicidal ideations (*17**,**18*). For 2 patients who died of pulmonary embolism, the committee decided that immobilization related to severe MDR TB disease predisposed them to pulmonary embolism and increased their risk of death. 

**Table 1:** Medical conditions related to underlying cause of death for tuberculosis patients, Karnataka, India*

Consensus reached from verbal autopsy	Consensus reached after discussion
Breathlessness	HIV-related opportunistic infections
Chest pain	Acute myocardial infarction
Hemoptysis	Hypertensive heart disease
Cardiac arrest	History of cardiac enlargement
Productive cough	Bilateral foot edema suggestive of congestive cardiac failure
Loss of appetite	Hemorrhagic shock and anemia
History of stroke	Alcoholic gastritis
Mental confusion	Alcoholic liver disease
Death by hanging	Hepatic failure
Drowning	Sepsis due to tuberculosis
Acid consumption	Unilateral feet edema suggestive of pulmonary embolism
HIV/TB co-infection	Oral cancer

*A 3-member commission determined underlying cause of death after verbal autopsy (interviews and record review), or after in-person discussion if the members could not reach consensus.

**Table 2:** Underlying cause of death for patients who died during treatment for multidrug-resistant tuberculosis, Karnataka, India*

Underlying cause of death (ICD-10 code)	No. patients, n = 72
Infectious diseases (A00–B99)	
Tuberculosis (A15)*	55
Diarrhea and gastroenteritis of presumed infectious origin (A09)	2
HIV disease resulting in infectious and parasitic infection (B20)	3
Neoplasms (C00–D48)	
Malignant neoplasm of other and unspecified parts of tongue (C02)	1
Disease of the nervous system (G00–G99)	
Hemiplegia (G81)	1
Diseases of the circulatory system (I00–I99)	
Hypertensive heart failure (I11)	1
Acute myocardial infarction (I21)	1
Hypotension (I95)	1
Diseases of the digestive system (K00–K93)	
Alcoholic gastritis (K29.2)	1
Peptic ulcer, site unspecified (K27)	1
Alcoholic liver disease (K70)	3
Acute hepatic failure, not otherwise specified (K72)	1
Symptoms, signs, and abnormal clinical and laboratory finding, not elsewhere classified (R00–R99)	
Other ill-defined and unspecified causes of mortality (R99)	1

*Underlying cause of death was assigned as TB if it had initiated the sequence of morbid events leading directly to death. ICD-10, International Classification of Diseases, 10th Revision.

We described the characteristics of the 71 MDR TB patients who died during treatment by underlying cause of death (Table 3). The mean age of the patients was 44 years (SD ± 14 years), and more than two thirds (69%) were male. Deaths with TB as the underlying cause were significantly less common (p = 0.011) among male patients (Table 3). The median (IQR) duration of treatment for patients with TB as underlying cause of death was 192 (101–365) days and for those without was 158 (76–459) days. The median (IQR) number of missed TB doses for patients with TB as underlying cause of death was 24 (6–60) and for those without was 20 (9–46). We saw no significant differences in the duration of treatment (p = 0.50) or the number of missed doses (p = 0.16) between patients with TB as underlying cause of death and those without.

**Table 3:** Characteristics associated with death in patients who died during treatment for MDR TB, Karnataka, India*

Characteristic	No. (%) for whom TB is the underlying cause of death	No. (%) for whom TB is not the underlying cause of death	χ^2^ test result (p value)†
Total, n = 71	55 (77)	16 (23)	
Age, y			
15–30	9 (90)	1 (10)	3.06 (0.383)
31–45	22 (82)	5 (18)	
46–60	16 (76)	5 (24)	
61–75	8 (62)	5 (38)	
Sex			
M	33 (69)	15 (31)	6.44 **(0.011)**
F	22 (96)	1 (4)	
Socioeconomic status			
Above poverty line	5 (63)	3 (37)	1.49 (0.474)
Below poverty line	42 (81)	10 (19)	
Not available	8 (73)	3 (27)	
Smoking history			
Yes	20 (67)	10 (33)	3.73 (0.155)
No	33 (85)	6 (15)	
Don’t know	2 (100)	0	
Alcohol consumption history			
Yes	18 (64)	10 (36)	4.71 (0.095)
No	36 (86)	6 (14)	
Don’t know	1 (100)	0	
Diabetes			
Yes	11 (79)	3 (21)	0.012 (0.912)
No	44 (77)	13 (23)	
Hypertension			
Yes	11 (79)	3 (21)	0.012 (0.912)
No	44 (77)	13 (23)	

*The data do not include 1 patient whose underlying cause of death could not be ascertained. MDR, multidrug-resistant; TB, tuberculosis. †Bold indicates statistically significant p values (p<0.05).

## Discussion

Our methods were consistent with the current national policy in India of conducting death audits by public health authorities to ascertain the cause of death and identify gaps in program implementation (*19*). We were not aware of previous reports of using verbal autopsy to ascertain the underlying cause of death among patients treated for MDR TB in India. Death rates among MDR TB patients treated in Maharashtra and Western India ranged 21%–30% (*20**,**21*). These reports defined TB-related deaths in accordance with the RNTCP treatment outcome variable (i.e., all-cause mortality) and may not reflect the true estimate of deaths attributable to TB. A recent study among a smaller cohort of HIV-infected MDR TB patients treated in Mumbai reported 4 (31%) of 13 deaths were attributed to causes not related to TB or treatment (*22*). These data suggest that the RNTCP may be overestimating TB-related deaths among MDR TB patients. In our study, we found that 23% (16/71) of patients who died during MDR TB treatment did not have TB as an underlying cause of death.

Our study had several strengths. We identified all TB patients in the national control program who died during the study period and had a high proxy response rate (96%). Therefore, selection bias was unlikely. In contrast to using the death audit method, which is not well-defined (*19*), we systematically gathered the medical history of patients who had died by interviewing key informants and reviewing medical records and death certificates. These additional sources of information increased the total data available for each case that might otherwise be missed by reviewing medical records alone. Furthermore, we constituted an independent committee of medical specialists not affiliated with RNTCP to review all the available evidence and code the underlying causes of death, which reduced bias that may occur when TB program personnel conduct a death audit. Consensus among committee members increased the validity of our results. In addition, causes of death secondary to TB, such as treatment-related suicide and pulmonary embolism (*23*), enriched the qualitative aspects of TB-related deaths that could otherwise be missed.

Our study had some limitations. In India, as many as 2 million TB patients seek care in the largely unregulated private sector (*24*). We included only patients who received treatment from the national TB control program; thus, our study findings do not reflect all MDR TB patients in India. Unlike the RNTCP death audit, which focuses on identifying potential gaps in program implementation (*19*), our study focused only on ascertaining the medical cause of death. Therefore, we are unable to comment on the potential gaps in program implementation that may have precipitated death. Furthermore, as with all verbal autopsy studies, the robustness of the results depends on the accuracy of the information provided by key informants. We worked to minimize errors by following standard procedures, but it is possible that some errors may have occurred. We did not collect information on whether the deceased patients had undergone drug susceptibility testing for the second-line TB drugs and so are unable to comment if persons with more severe forms of TB disease were more likely to die of TB than those with less severe forms of TB.

Despite these limitations, the study has several implications for policy and practice. First, a substantial proportion of deaths during MDR TB treatment were not TB related. Our results suggest the national TB program may be overestimating TB case-fatality rates. Incorporating verbal autopsies as part of the death audit may help improve the accuracy of defining TB-related deaths. Second, suicide was common (n = 6) and attributed as secondary to TB disease or treatment of TB. These deaths were potentially preventable. Cycloserine is known to lead to psychiatric adverse reactions, including suicidal tendencies (*2**,**17*). WHO recently recommended that cycloserine be replaced with other oral second-line drugs (*25*). In addition, 4 patients had alcohol-related deaths. While these deaths were not considered secondary to TB, it is unclear if these patients also had depression. A population-based study in Kerala reported higher risk for poor treatment outcome, including death, among MDR TB patients consuming alcohol (*26*). These data reinforce the need for professional counseling and psychiatric care integration for MDR TB care (*2*). A recent study assessed the feasibility of integrated psychiatric and medical TB care and treatment and suggested the immediate need in India (*27*). Third, our finding that male sex was associated with deaths due to causes other than TB may be helpful in generating hypotheses for further research, such as risk-factor analysis in a larger, more representative cohort of patients throughout India. Finally, we acknowledge that many TB-related deaths may occur before the start of MDR treatment, after completion of MDR treatment, and among persons lost to follow-up. We are hopeful our findings will stimulate further research to document all potential TB-related deaths in the community and aid in monitoring India’s progress toward reducing TB deaths by 95% by 2035 (*28*).

Technical Appendix Additional information about the verbal autopsy method used for study of underlying cause of death during treatment of multidrug-resistant tuberculosis, India.
