# Supplementary material for: Use of Verbal Autopsy to Determine Underlying Cause of Death during Treatment of Multidrug-Resistant Tuberculosis, India
Source: Emerg Infect Dis. 2018 Mar;24(3):478–84. doi: 10.3201/eid2403.171718 (PMC5823351; doi:10.3201/eid2403.171718)
Supplement: Technical Appendix — Additional information about the verbal autopsy method used for study of underlying cause of death during treatment of multidrug-resistant tuberculosis, India. [file 17-1718-Techapp-s1.pdf]

Article DOI: https://doi.org/10.3201/eid2403.171718

Use of Verbal Autopsy to Determine Underlying Cause of Death during Treatment of Multidrug-Resistant Tuberculosis, India

Technical Appendix

Verbal Autopsy and Death Registration Details Abstraction Instrument

DR TB Center:

District the patient belongs to:

DOTS PLUS TB no.:

Date of registration:

Date of death:

Unique ID No.:

Name of the Investigator:

Date of interview: (dd/mm/year):

Name of the deceased: (This information will be delinked from the main data):

Name of the respondent and relation with the deceased: (This information will be delinked from the main data):

Contact number of the respondent: (This information will be delinked from the main data):

Address of the respondent: (This information will be delinked from the main data):

Variables	Categories	Write / Tick the appropriate responses
What is your relationship to the deceased?	1. Father<br>2. Mother<br>3. Spouse<br>4. Sibling<br>5. Other relative	

Variables	Categories	Write / Tick the appropriate responses
	(specify)<br>6. No relation	
Gender of the deceased	1. Male<br>2. Female	
Age of the deceased at death	______ (Years) months ______	
Did you live with the deceased in the period leading to her/his death and for how long you were with the deceased??	1. Yes<br>2. No	
When did s/he die? (dd/mm/yy)		
What was the site of death?	1. Hospital<br>2. Other Health Facility<br>3. Home<br>4. Other (Specify)<br>5. Don't Know	
What was her/his occupation, that is, what kind of work did s/he mainly<br>do?	Mention	
Type of ration card	APL/ BPL/ No card	
**Context and History Of Previously Known Medical Conditions**		
Was the patient taking tuberculosis treatment?	Yes	
	No	
	Don’t know	
For how long?	Number of Days<br>Weeks<br>Months	
From where the treatment was being taken?	Specify which type of health care facility	
Was malaria diagnosed?	Yes	
	No	
	Don’t know	
Was high blood pressure diagnosed? (If yes specify duration in months/ years)	Yes	
	No	
	Don’t know	
Was heart disease diagnosed? (If yes specify duration in months/ years)	Yes	
	No	
	Don’t know	
Was stroke diagnosed? (If yes specify duration in months/ years)	Yes	
	No	
	Don’t know	
Was diabetes diagnosed? (If yes specify duration in months/ years)	Yes	
	No	
	Don’t know	
Was any type of cancer diagnosed?<br>(If yes, specify type and specify duration in months/ years)	Yes	
	No	
	Don’t know	
Was any mental illness diagnosed?<br>(If yes, specify type and specify duration in months/ years)	Yes	
	No	
	Don’t know	
Was liver disease diagnosed? (If yes specify duration in months/ years)	Yes	
	No	
	Don’t know	
Was kidney disease diagnosed? (If yes specify duration in months/ years)	Yes	
	No	
	Don’t know	
Was she pregnant? (This question is applicable only if the deceased is female)	Yes	
	No	
	Don’t know	
Did he/she suffer from any injury or accident?	Yes	
	No	
	Don’t know	
If yes, what type of injury or accident was it?	Explain it	
If yes, was it severe enough to cause hospitalization or lead to death?	Yes	
	No	
	Don’t know	

Variables	Categories	Write / Tick the appropriate responses
Any other specific cause leading to death as per the kin of the respondent?	Yes	
	No	
	Don't know	
If yes for the above question, mention the cause		
**Symptoms Noted During the Final Illness**		
Did s/he have fever?	Yes	
	No	
	Don't know	
For how long did s/he have fever?	<2 weeks preceding death	
	≥2 weeks preceding death	
Did s/he have fever with chills or rigors?	Yes	
	No	
	Don't know	
For how long did s/he have fever with chills or rigors?	<2 weeks preceding death	
	≥2 weeks preceding death	
Did s/he have cough?	Yes	
	No	
	Don't know	
For how long did s/he have cough?	<2 weeks preceding death	
	≥2 weeks preceding death	
Did s/he have cough with purulent sputum?	Yes	
	No	
	Don't know	
For how long did s/he have cough with purulent sputum?	<2 weeks preceding death	
	≥2 weeks preceding death	
Did s/he have cough with hemoptysis?	Yes	
	No	
	Don't know	
For how long did s/he have cough with hemoptysis?	<2 weeks preceding death	
	≥2 weeks preceding death	
Did s/he have night sweats?	Yes	
	No	
	Don't know	
For how long did s/he have night sweats?	<2 weeks preceding death	
	≥2 weeks preceding death	
Did s/he have any breathing problem?	Yes	
	No	
	Don't know	
Did s/he have fast breathing?	Yes	
	No	
	Don't know	
For how long did s/he have fast breathing?	<2 weeks preceding death	
	≥2 weeks preceding death	
Did s/he have breathlessness?	Yes	
	No	

Variables	Categories	Write / Tick the appropriate responses
	Don't know	
For how long did s/he have breathlessness?	<2 weeks preceding death	
	≥2 weeks preceding death	
Did s/he have severe chest pain?	Yes	
	No	
	Don't know	
For how long did s/he have chest pain?	<2 weeks preceding death	
	≥2 weeks preceding death	
Did s/he have diarrhea?	Yes	
	No	
	Don't know	
For how long did s/he have diarrhea?	<2 weeks preceding death	
	≥2 weeks preceding death	
At any time during the final illness was there blood in the stools?	Yes	
	No	
	Don't know	
At any time during the final illness did he/she have vomiting?	Yes	
	No	
	Don't know	
Did s/he vomit "coffee grounds" or bright red/blood?	Yes	
	No	
	Don't know	
Did s/he have severe abdominal pain?	Yes	
	No	
	Don't know	
For how long did s/he have abdominal pain?	<2 weeks preceding death	
	≥2 weeks preceding death	
Did s/he have more than usual protruding abdomen?	Yes	
	No	
	Don't know	
For how long did s/he have a more than usual protruding abdomen?	<2 weeks preceding death	
	≥2 weeks preceding death	
Did s/he have severe headache?	Yes	
	No	
	Don't know	
For how long did s/he have severe headache?	<2 weeks preceding death	
	≥2 weeks preceding death	
Did s/he have a stiff or painful neck?	Yes	
	No	
	Don't know	
For how long did s/he have a stiff or painful neck?	<2 weeks preceding death	
	≥2 weeks preceding death	
Did s/he have mental confusion at any time during the final illness?	Yes	
	No	
	Don't know	
Was s/he unconscious for more than 24 h?	Yes	
	No	
	Don't know	
Did s/he have fits?	Yes	
	No	
	Don't know	
Did s/he have any urine problems?	Yes	
	No	
	Don't know	
Did s/he pass no urine at all at any time during the final illness?	Yes	
	No	
	Don't know	
During the final illness did s/he ever pass blood in the urine?	Yes	
	No	
	Don't know	
Did s/he have weight loss?	Yes	
	No	
	Don't know	

Variables	Categories	Write / Tick the appropriate responses
Was s/he severely thin or wasted?	Yes	
	No	
	Don’t know	
Did s/he have mouth sores or white patches in the mouth or on the tongue?	Yes	
	No	
	Don’t know	
Did s/he have any skin problems?	Yes	
	No	
	Don’t know	
Did s/he have swelling (puffiness) of the face?	Yes	
	No	
	Don’t know	
For how long did s/he have swelling (puffiness) of the face?	<2 weeks preceding death	
	≥2 weeks preceding death	
Did s/he have both feet swollen?	Yes	
	No	
	Don’t know	
For how long did s/he have swelling of both feet?	<2 weeks preceding death	
	≥2 weeks preceding death	
Did s/he have any lumps on the neck?	Yes	
	No	
	Don’t know	
Did s/he have any lumps on the armpit?	Yes	
	No	
	Don’t know	
Did s/he have paralysis of one side of the body?	Yes	
	No	
	Don’t know	
For how long did s/he have paralysis of one side of the body?	<2 weeks preceding death	
	≥2 weeks preceding death	
Did s/he have yellow discoloration of the eyes?	Yes	
	No	
	Don’t know	
Did s/he look pale (thinning/lack of blood) or have pale palms, eyes or nail beds?	Yes	
	No	
	Don’t know	
Did s/he have (or needed) an operation for the illness?	Yes	
	No	
	Don’t know	
Did s/he have the operation within 1 mo before death?	Yes	
	No	
	Don’t know	
Did s/he drink alcohol?	Yes	
	No	
	Don’t know	
Did s/he smoke tobacco (cigarette, cigar, pipe, etc.)?	Yes	
	No	
	Don’t know	
For how long was s/he ill before s/he died?	Don’t know	
	Days	
	Weeks	
	Months	
Care received for the last illness	Yes	
	No	
	Don’t know	

Description of the illness by the respondent:

1. Chronology of events that lead to death: (Description as per the respondent: The most recent condition featuring first and the earliest, i.e., the condition that started the sequence of events between normal health and death featuring last.)

2. Causes of death as per the respondent:

i.

ii.

Information recorded in the medical records available with the family:

Any co-morbidities:

Diagnosis:

Any other significant information pertaining to medical/ surgical or lifestyle:

Information recorded in the death certificate available with the family:

Disease or condition directly leading to death:

Morbid conditions if any giving rise to the above cause:

Other significant conditions contributing to death:

Development and Finalization of the Tool

Five physicians rated available evidence (verbal autopsy tool) using a three-point Likert scale consisting of essential, important but not essential, and neither essential nor important. In addition, qualitative responses improved interpretability of some variables. If all 5 subject experts rated an item as essential (100% agreement) it was retained. All items were rated as essential under the “baseline characteristics” section of the questionnaire. All items were also rated as essential under the sections “context and history of previously known medical conditions,” “description of the illness by the respondent,” “medical records available with the family,” and “death certificate available with the family.” Under the section “symptoms noted during the final illness,” the word “convulsions” was replaced by “fits” for better comprehension of the field investigators. Two additional questions on cough with purulent sputum and fever with chills and rigors were added to rule out specific morbidities. The questionnaire was then used in the field.
